# XPO1 inhibitor combination therapy with bortezomib or carfilzomib induces nuclear localization of IκBα and overcomes acquired proteasome inhibitor resistance in human multiple myeloma

**DOI:** 10.18632/oncotarget.12969

**Published:** 2016-10-28

**Authors:** Joel G. Turner, Trinayan Kashyap, Jana L. Dawson, Juan Gomez, Alexis A. Bauer, Steven Grant, Yun Dai, Kenneth H. Shain, Mark Meads, Yosef Landesman, Daniel M. Sullivan

**Affiliations:** ^1^ Chemical Biology and Molecular Medicine Program, H. Lee Moffitt Cancer Center and Research Institute, Tampa, FL, USA; ^2^ Karyopharm Therapeutics, Natick, MA, USA; ^3^ Massey Cancer Center, Virginia Commonwealth University, Richmond, VA, USA; ^4^ Department of Malignant Hematology, H. Lee Moffitt Cancer Center and Research Institute, Tampa, FL, USA; ^5^ Department of Blood & Marrow Transplantation, H. Lee Moffitt Cancer Center and Research Institute, Tampa, FL, USA

**Keywords:** XPO1, bortezomib, carfilzomib, multiple myeloma, acquired drug resistance

## Abstract

Acquired proteasome-inhibitor (PI) resistance is a major obstacle in the treatment of multiple myeloma (MM). We investigated whether the clinical XPO1-inhibitor selinexor, when combined with bortezomib or carfilzomib, could overcome acquired resistance in MM. PI-resistant myeloma cell lines both *in vitro* and *in vivo* and refractory myeloma patient biopsies were treated with selinexor/bortezomib or carfilzomib and assayed for apoptosis. Mechanistic studies included NFκB pathway protein expression assays, immunofluorescence microscopy, ImageStream flow-cytometry, and proximity-ligation assays. IκBα knockdown and NFκB activity were measured in selinexor/bortezomib-treated MM cells. We found that selinexor restored sensitivity of PI-resistant MM to bortezomib and carfilzomib. Selinexor/bortezomib treatment inhibited PI-resistant MM tumor growth and increased survival in mice. Myeloma cells from PI-refractory MM patients were sensitized by selinexor to bortezomib and carfilzomib without affecting non-myeloma cells. Immunofluorescence microscopy, Western blot, and ImageStream analyses of MM cells showed increases in total and nuclear IκBα by selinexor/bortezomib. Proximity ligation found increased IκBα-NFκB complexes in treated MM cells. IκBα knockdown abrogated selinexor/bortezomib-induced cytotoxicity in MM cells. Selinexor/bortezomib treatment decreased NFκB transcriptional activity. Selinexor, when used with bortezomib or carfilzomib, has the potential to overcome PI drug resistance in MM. Sensitization may be due to inactivation of the NFκB pathway by IκBα.

## INTRODUCTION

Cancer cells utilize the process of nuclear-cytoplasmic transport through the nuclear pore complex to effectively evade anti-cancer mechanisms [1–5]. We have shown that knockdown of exportin 1 (XPO1/CRM1) protein by siRNA or with an XPO1 inhibitor will sensitize drug-resistant myeloma cells to the topoisomerase II (TOP2) inhibitor doxorubicin [3, 5]. In addition, we found that XPO1 inhibitors are able to prevent nuclear export and promote nuclear accumulation of the tumor suppressor protein p53 [3, 5]. XPO1 inhibitors, when used in combination with the proteasome inhibitors (PI) bortezomib and carfilzomib, were found to synergistically kill multiple myeloma (MM) cells and when co-cultured with bone marrow stromal cells [5, 6]. Our studies have shown that MM patient bone marrow mononuclear cells, when co-treated with an XPO1 inhibitor and PIs, synergistically induced apoptosis in MM cell populations but not in non-myeloma bone marrow cells, indicating that XPO1 inhibition may specifically inhibit cancer cells in MM patients [4, 5]. These studies were the first to report cancer cell-specific apoptosis by combinations of XPO1 with PI. However, acquired drug-resistance results in cell lines, *in vitro* and *in vivo*, and ex vivo in PI-refractory patients have not been investigated in MM.

Recent publications have indicated that XPO1 inhibitors, especially the orally available clinical compound selinexor (KPT-330), may be effective against various hematologic malignancies, including leukemia [7–12], mantle cell lymphoma [13, 14], and MM [5, 10, 15].

High levels of XPO1 may be associated with decreased event-free and overall survival in MM [10]. Recent studies in MM have shown that XPO1 protein levels are increased in plasma cells from newly diagnosed MM patients compared with normal plasma cells [10, 15] or with plasma cells from those with monoclonal gammopathy of undetermined significance and smoldering MM [15]. XPO1 mRNA is also increased in bortezomib-treated patient samples [10]. In addition, when treated with XPO1 inhibitors, 21 different human MM cell lines were found to have decreased cell viability [3, 5, 10, 15]. XPO1 inhibitors in human MM have been shown to inhibit the export of the following cancer-related proteins or mRNAs from the nucleus: c-myc, CDC25A, BRD4, p53, Mcl-1, BCl-xL, NFκB, p21, p27, IκB, FOXO3A, FOXO1A, PP2A, PUMA, BAX, CHOP, C1-0orf10, MIC1, IL-6, VEGF, MIP1ß, and IL-10 [5, 10, 15].

What has not been addressed in previous studies is whether XPO1 inhibitors are effective in overcoming acquired drug-resistant MM phenotypes, which develop in patients during treatment with PIs. In patients with MM, drug resistance is the primary limitation to successful treatment. Myeloma is still considered incurable despite significant advances afforded by immunomodulatory drugs (thalidomide, lenalidomide, pomalidomide), PIs (bortezomib, carfilzomib, ixazomib), antibodies targeting SLAMF7 protein (elotuzumab) and CD38 (daratumumab), histone deacetylase inhibitors (panobinostat), and high-dose melphalan with autologous stem cell rescue.

In the present study, we show that XPO1 inhibition sensitized acquired PI-resistant MM cells to bortezomib and carfilzomib in both *in vitro* and *in vivo* models and *ex vivo* in PI-refractory patient CD138+/light chain+ MM cells, thus showing that this combination may provide a means to overcoming acquired drug resistance in MM.

## RESULTS

### XPO1 inhibition sensitizes PI-resistant MM cell lines to bortezomib and carfilzomib

**Figure 1:**
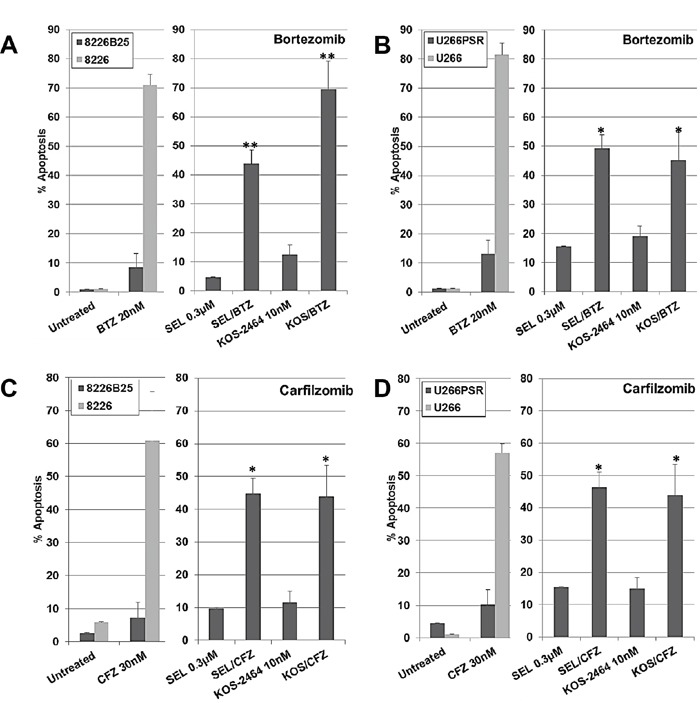
XPO1 inhibition sensitizes PI-resistant human multiple myeloma (MM) cell lines to bortezomib (BTZ) and carfilzomib (CFZ) Human U266 **B/D.** and 8226 **A/C.** drug-resistant and parental MM cell lines were treated concurrently for 20 h with selinexor (300 nM) or KOS-2464 (10 nM) +/− BTZ (20 nM) or +/− CFZ (30 nM) and assayed for apoptosis by flow cytometry (activated caspase 3). Resistant MM cell lines were up to 10-fold resistant to single-agent BTZ or CFZ compared with parental cells. The addition of the XPO1 inhibitors selinexor (SEL) or KOS-2464 sensitized drug-resistant cells to BTZ or CFZ compared with single-agent BTZ or CFZ (*p = 0.0054, **p = 0.0017). All cells were grown at log-phase growth conditions (5×10^5^ cells/mL).

Apoptosis results (flow cytometry using activated caspase 3) from human PI-resistant and parental MM cells after 20-hour concurrent treatment with selinexor (300 nM) or KOS-2464 (10 nM) ± bortezomib (10 nM) or carfilzomib (20 nM) are shown in Figure 1. Both U266 and 8226 parental cell lines were highly sensitive to single-drug treatment with bortezomib or carfilzomib at log-phase growth densities (5 × 10^5^ cells/mL). PI-resistant U266PSR and 8226B25 MM cell lines [16, 17] were resistant to single-agent bortezomib (up to 10-fold) or carfilzomib (up to 9-fold) when compared to parental cells (Figure 1). When the XPO1 inhibitor selinexor was added, both U266PSR and 8226B25 PI-resistant cells were highly sensitized to bortezomib (*P* = 0.00055 and *P* = 0.0054, respectively) or carfilzomib (*P* = 0.0017 and *P* = 0.0033, respectively) treatment compared with single-agent treatment (Figure 1). Equivalent results were found when PIs were used with the XPO1 inhibitor KOS-2464 [18] (Figure 1).

### *In vivo* NOD/SCID-γ mouse studies with selinexor and bortezomib

**Figure 2:**
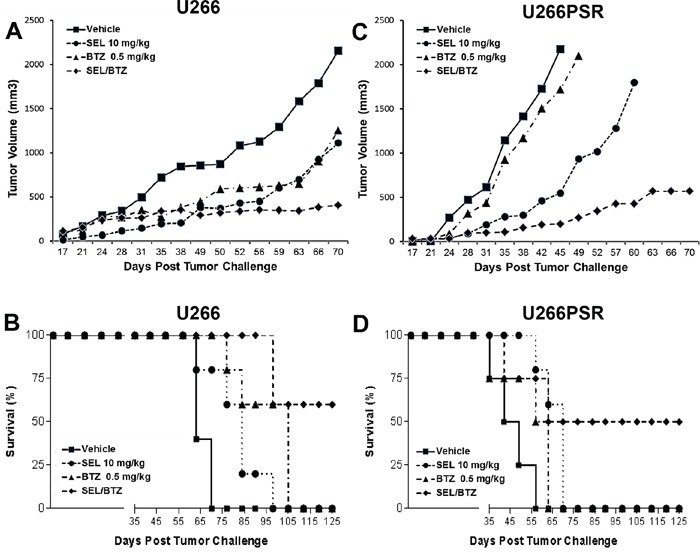
NOD/SCID-γ (NSG) mouse *in vivo* studies NSG mice (n=5 per group) were challenged subcutaneously with 10^7^ U266 (A/B) or 10^6^ proteasome inhibitor (PI)-resistant U226PSR (C/D) human MM cells. Mice were treated twice weekly (Monday, Thursday) with selinexor +/− BTZ. selinexor was administered by oral gavage and BTZ by intraperitoneal injection. **A/C.** Tumor growth with selinexor and BTZ. BTZ/selinexor combination reduced tumor growth compared with single-agent BTZ (*P* = 0.022) or vehicle control (*P* = 0.0014). **B/D.** Survival with selinexor and BTZ. In NSG mice challenged with U266 tumors, selinexor/BTZ treatment improved survival compared with vehicle (*P* = 0.0006) or single-agent selinexor (*P* = 0.0010) or BTZ (*P* = 0.0072). Treatment of PI-resistant PSR tumors with selinexor/BTZ also improved survival compared with vehicle control (*P* = 0.0001) and single-agent BTZ (*P* = 0.0001) or selinexor (*P* = 0.0085). Toxicity, assessed by weight loss (<10%), was minimal in all treatment groups.

In our mouse studies, we used both PI-resistant (U266PSR) and parental U266 human MM cells. U266PSR cells have been shown to be up to 10-fold resistant to bortezomib and up to 9-fold resistant to carfilzomib (Figure 1) [16, 17, 19]. As shown in Figure 2A, bortezomib combined with selinexor resulted in reduced U266 MM tumor growth versus single-agent bortezomib (*P* = 0.022), selinexor (*P* = 0.033), or vehicle control (*P* = 0.00051) (Figure 2A). NOD/SCID-γ mice challenged with PI-resistant U266PSR MM tumors also had reduced tumor growth with selinexor/bortezomib compared with single-agent bortezomib (*P* = 0.0006), selinexor (*P* = 0.018), or vehicle control (*P* = 0.0014) (Figure 2C). Combining bortezomib and selinexor improved survival in mice with U266 MM tumors compared with single-agent bortezomib (*P* = 0.0072), selinexor (*P* = 0.0010), or vehicle (*P* = 0.0006) (Figure 2B). Survival in mice with PI-resistant U266PSR tumors improved with selinexor/bortezomib treatment compared with single-agent bortezomib (*P* = 0.0072), selinexor (*P* = 0.0010), or vehicle (*P* = 0.0006) (Figure 2D). At the end of the study (125 days), 60% of U226 parental and 50% of U266PSR challenged mice treated with bortezomib and selinexor were tumor-free, all other treatment groups did not survive. Toxicity, assessed by weight loss, was minimal in all treatment groups.

### *Ex vivo* treatment of newly diagnosed, relapsed, and PI-refractory patient MM cells with selinexor and KOS-2464 sensitizes cells to bortezomib and carfilzomib

**Figure 3:**
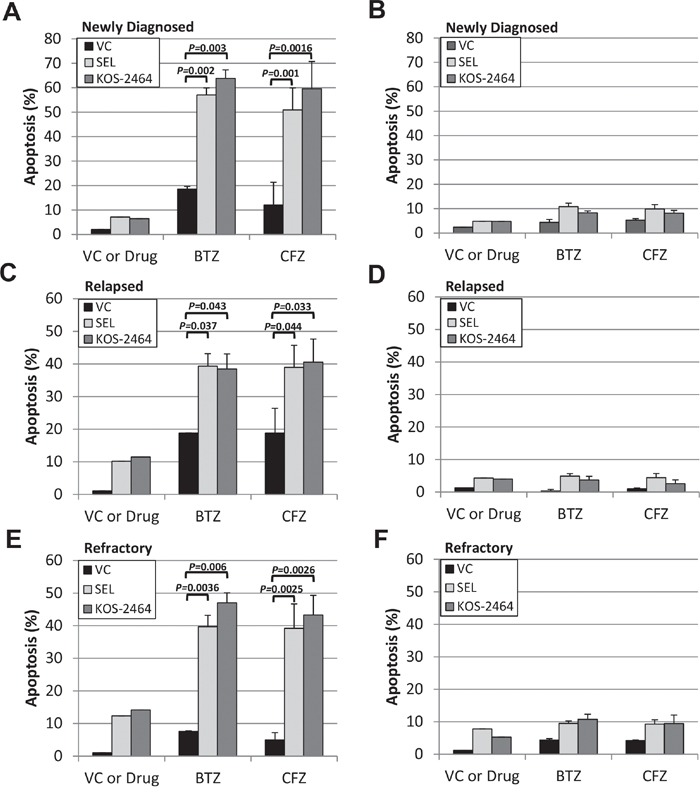
KOS-2464 and selinexor sensitize newly diagnosed, relapsed, and refractory patient MM cells to BTZ and CFZ Bone marrow mononuclear cells from myeloma patients were treated *ex vivo* with selinexor (300 nM) or KOS-2464 (300 nM) and BTZ (10 nM) or CFZ (20 nM). Apoptosis was assayed for activated caspase 3 by flow cytometry in cell populations that were positive for both CD138 and light-chain immunoglobulin (kappa or lambda). Newly diagnosed (n=8) **A.**, relapsed (n=5) **C.**, and BTZ (n=8)/CFZ (n=6) refractory **E.** MM patient samples were all sensitized by selinexor and KOS-2464 to BTZ (*P* = 0.0016 to 0.043, respectively) and CFZ (*P* = 0.001 to 0.044, respectively). CD138/light-chain double-negative patient cells were not sensitized to apoptosis by XPO1 inhibitors **B, D, and F.** VC, vehicle control.

Using flow cytometry, we gated on the CD138/light-chain immunoglobulin double-positive myeloma cell population in patient bone marrow aspirates. Apoptosis, as measured by activated caspase 3 expression showed that newly diagnosed (n=8), relapsed (n=5), and bortezomib (n=8)/carfilzomib (n=6) refractory MM patient samples were sensitized by selinexor and KOS-2464 to both bortezomib (*P* = 0.043 to 0.002) and carfilzomib (*P* = 0.044 to 0.001) (Figure 3A, 3C, and 3E). When gating on the CD138/light-chain double-negative non-myeloma cells, we found that they were not sensitized to apoptosis by XPO1 inhibitors (Figure 3B, 3D, and 3F). These data indicate that myeloma cells were targeted by the XPO1 inhibitor/PI drug combination and that non-myeloma cells were relatively unaffected.

### Selinexor-bortezomib combination treatment induced NFκB-IκBα complex formation

**Figure 4:**
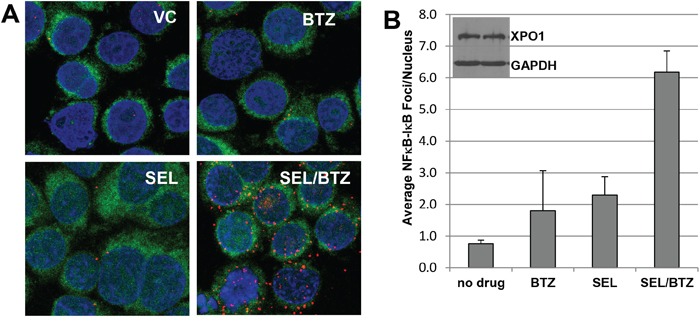
Selinexor promotes NFκB-IκBα binding **A.** Proximity ligation assay for 8226B25 PI-resistant MM cells (3×10^6^/ml) treated and stained with antibodies for NFκB and IκBα. Selinexor (KPT-330) in combination with BTZ increased proximity co-localization of NFκB and IκBα up to 12-fold over untreated and single-agent BTZ or selinexor. Green fluorescence denotes the cytoplasm, and blue indicates the nucleus (DAPI). **B.** Selinexor/BTZ significantly increased the number of NFκB-IκBα foci in the nucleus versus no drug or single-agent selinexor or BTZ (*P* ≤ 0.00077) (n=3, 50 cells per assay). **Inset:** Selinexor treatment did not affect XPO1 protein expression at 4 hours as shown by Western blot.

Proximity ligation assays (n=2) were performed as described under Materials and Methods. In this assay, an NFκB-IκBα complex formation will produce a red fluorescent signal when NFκB and IκBα are in close proximity (<40 nM). PI-resistant and parental cells treated for 6 hours with vehicle (1% DMSO), selinexor, or bortezomib as single agents produced very few NFκB-IκBα complexes; however, the combination of selinexor/bortezomib increased NFκB-IκBα co-localization 12-fold more than untreated cells and 10-fold and 5-fold more than single-agent bortezomib and selinexor, respectively (Figure 4A). Increased nuclear NFκB-IκBα binding may result in inactivation of NFκB transcriptional activity, decreased cell proliferation, and increased apoptosis (Figure 4B) [20–22].

### Selinexor-bortezomib increased IκBα protein expression as shown by immunofluorescence microscopy and Western blot

**Figure 5:**
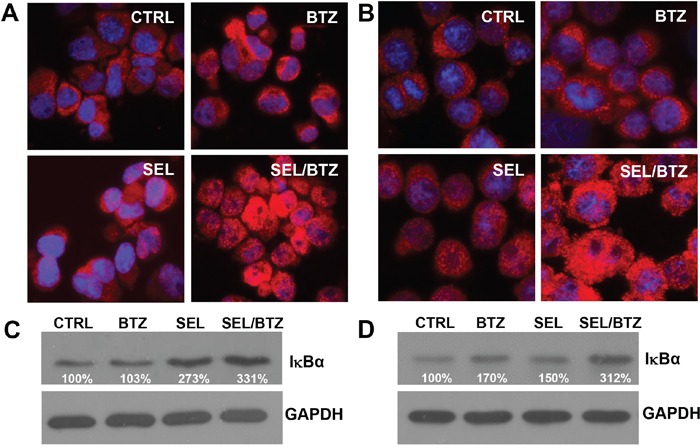
Immunofluorescence microscopy and Western blot of IκBα in PI-resistant MM cell lines **A/B.** Immunofluorescence microscopy, U266PSR (A) and 8226B25 (B) PI-resistant cells showed an increase in IκBα (red) after treatment with selinexor/BTZ compared with untreated control or single-agent BTZ or selinexor. **C/D.** Selinexor/BTZ combination treatment increased IκBα protein in U266PSR (331%) and 8226B25 (312%) cells compared with untreated control or single-agent BTZ or selinexor (n=4).

Immunofluorescence microscopy demonstrated an increase in IκBα protein in PI-resistant U266PSR (Figure 5A) and 8226B25 (Figure 5B) cells treated with selinexor in combination with bortezomib. Western blot confirmed these data in all cell lines tested. PI-resistant U226PSR and 8226B25 cells had increased IκBα protein (331% and 312%, respectively) in selinexor/bortezomib-treated cells compared with untreated cells (Figure 5C and 5D).

### Immunofluorescence microscopy of IκBα in patient MM treated with selinexor

**Figure 6:**
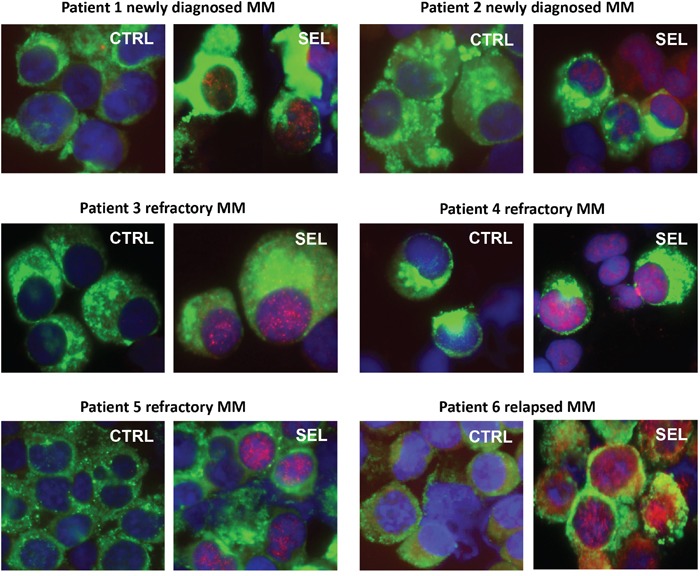
Immunofluorescence microscopy of IκBα in patient MM treated with selinexor Bone marrow aspirates from newly diagnosed, relapsed, and refractory MM patients were treated *ex vivo* with 100-300 nM selinexor for 20 hours. MM cells were identified by kappa/lambda light chain antigen staining (green), nuclei identified by DAPI (blue) staining, and IκBα protein (red). All patient MM samples showed an increase in IκBα protein, especially in the cell nuclei with *ex vivo* selinexor treatment compared with untreated controls from the same patient.

Bone marrow aspirates from newly diagnosed, relapsed, and refractory MM patients were treated *ex vivo* with selinexor (Figure 6). Kappa/lambda light chain antigen-positive MM cells had increased IκBα protein staining, especially in the cell nuclei with *ex vivo* selinexor treatment compared with untreated controls from the same patient.

### ImageStream flow cytometry shows an increase in nuclear IκBα with drug treatment

**Figure 7:**
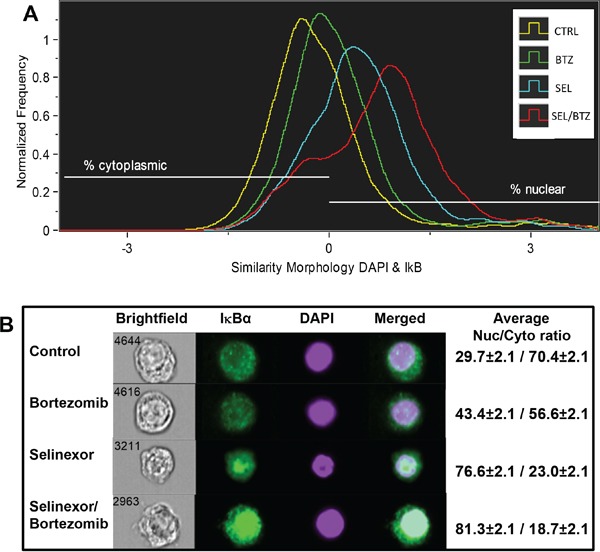
ImageStream flow cytometry **A.** 8226B25 human MM cells were treated for 20 hours with BTZ, SEL, or SEL/BTZ. An Imagestream histogram was generated using the similarity feature showing co-localization of IκBα (green) to the nuclear stain, DAPI (purple). IκBα shifted from the cytoplasm to the nucleus in BTZ-treated cells (*P* = 0.006), further shifted in SEL-treated cells compared with non-treated controls (*P* = 0.000067). However, the largest nuclear shift was seen in the SEL/BTZ combination treatment (n = 3) (*P* = 0.000057). **B.** Images of representative cells in real time, visually showing increased IκBα protein in the cell nuclei. Far right column shows the IκBα nuclear/cytoplasmic ratio for 5000 cells from each treatment group (n =3). *The similarity feature is the log-transformed Pearson correlation coefficient and is a measure of the degree to which two images are linearly correlated within a masked region.

8226B25 human MM cells treated for 20 hours with bortezomib, selinexor, or selinexor/bortezomib were analyzed by ImageStream flow cytometry (Figure 7). A histogram of nuclear and cytoplasmic IκBα showed that the percentage of total cellular IκBα in untreated cells was 29.7% nuclear and 70.4% cytoplasmic. Bortezomib treatment increased nuclear IκBα to 43.4% (*P* = 0.006) and selinexor treatment to 76.6% (*P* = 0.000067), with the largest nuclear shift found in the selinexor/bortezomib combination (81.3%) (*P* = 0.000057). Five thousand cells per treatment group were analyzed (Figure 7A) (n = 3). Images of representative cells in real time show increased IκBα protein in the cell nuclei with selinexor/bortezomib treatment (Figure 7B).

### Bortezomib-selinexor synergy may be linked to increased IκBα expression and subsequent down-regulation of NFκB transcriptional activity

**Figure 8:**
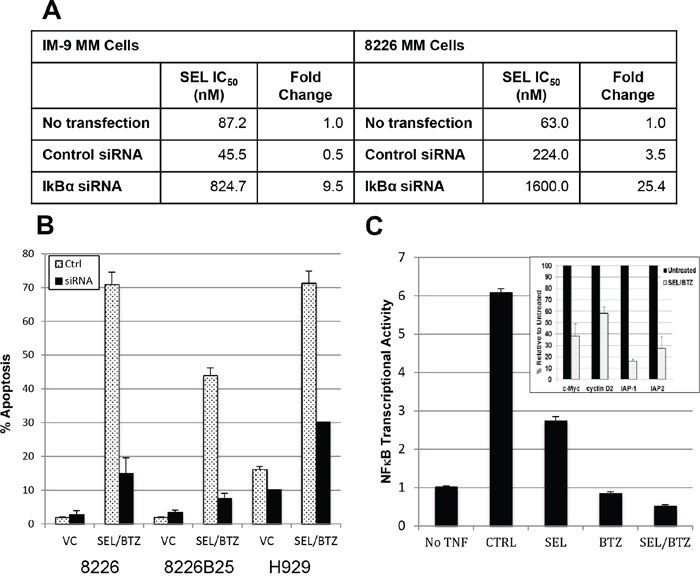
BTZ/selinexor synergy may be linked to IκBα expression and downregulation of NFκB transcriptional activity **A.** IM-9 and 8226 MM cells were transfected with 40 nM siRNA against IκBα or control siRNA. 24 hours posttransfection, the cells were treated with selinexor for 72 hours and IC_50_ determined. IκBα knockdown reduced toxicity to KPT-330 compared with control siRNA in both IM-9 (*P* = 0.023) and 8226 (*P* = 0.0062) cells. **B.** 8226, 8226B25, and H929 human MM cells were transfected with 40 nM IκBα siRNA. 48 hours after transfection, scram siRNA control and IκBα siRNA knockdown cells were treated with 100 nM selinexor ± BTZ. IκBα knockdown reduced apoptosis (activated caspase 3) in selinexor/BTZ-treated cells (*P* = 0.0086) compared with control siRNA. **C.** Chemiluminescent transcription factor assay. MM.1S cells were pretreated with 1 μM selinexor ± 100 nM BTZ for 2 hours and then exposed to 20 ng/mL of TNFα for 4 hours in serum-free media. TNFα exposure induced NFκB transcriptional activity 6-fold. Single-agent selinexor (*P* = 0.000023) and BTZ (*P* = 0.00024) lowered NFκB transcriptional activity, and the combination of selinexor and BTZ further reduced the activity to 3-fold below baseline (*P* = 0.00013) (no TNFα). **(Inset)** Western blots of protein from 8226 selinexor/bortezomib-treated cells showed a decrease in NFκB-mediated IAP-1 (84%), IAP-2 (72.8%), c-Myc (62%), and cyclin D2 (42%) protein expression compared with untreated controls. Protein loading was confirmed by GAPDH expression.

SiRNA knockdown of IκBα in IM-9 and 8226 MM cells produced a 9.5-fold (*P* = 0.023) and 25.4-fold (*P* = 0.0062) increase in selinexor IC_50_ values, making these cells less sensitive to selinexor (Figure 8A). IκBα knockdown was > 60% at 24 hours following transfection. IκBα knockdown also highly reduced apoptosis (activated caspase 3) in selinexor/bortezomib-treated cells (*P* = 0.0086) compared with control siRNA (Figure 8B). MM.1S cells treated with selinexor/bortezomib combined and as single agents were assayed for transcriptional activity. Single-agent selinexor or bortezomib treatment lowered NFκB transcriptional activity 2- and 5-fold, respectively (*P* = 0.0000023 and 0.00024); however, when selinexor and bortezomib were used together, transcriptional activity was reduced 12-fold compared with TNFα controls (*P* = 0.000019) and 3-fold below the baseline (*P* = 0.00013) (no TNFα) activity, as measured by chemiluminescent transcription assay (Figure 8C). In addition, Western blots to measure NFκB transcriptional activity showed a decrease in the anti-apoptotic proteins IAP-1 (84%) and IAP-2 (72.8%) and cell cycle (proliferation) proteins c-myc (62%) and cyclin D2 (42%) in selinexor/bortezomib-treated cells compared with untreated controls (inset in Figure 8C). Protein loading was confirmed by GAPDH expression.

## DISCUSSION

Our findings show that XPO1 inhibitors, when used in combination with PIs, are highly effective against acquired PI-resistant human MM in *in vitro* assays with PI-resistant cell lines, *in vivo* in mice challenged with PI-resistant MM tumors, and *ex vivo* in patient MM cells that are refractory to PIs. These data strongly suggest that selinexor, when combined with PIs, may be an effective therapy for PI-resistant MM.

Previous studies have shown that IκBα is a tumor suppressor that dimerizes with NFκB and prevents its transcriptional activity, thus decreasing NFκB-driven proliferation and increased apoptosis in MM cells [20, 23]. Proteasome inhibitors have been shown to inhibit NFκB activity through stabilization of IκBα [21]. In addition XPO1 inhibition by leptomycin B has been shown to sequester NFκB-IκBα complexes in the nucleus. These complexes are unable to bind DNA in the nucleus and activate NFκB transcriptional activity [22]. We found that the combination treatment of an XPO1 inhibitor (selinexor) and a PI (bortezomib) synergistically increased IκBα more than single-agent selinexor or bortezomib treatment in both parental and PI-resistant MM cell lines and in patient myeloma cells. This observed synergistic increase in IκBα expression by selinexor and bortezomib resulted in a strong anti-tumor effect. Proximity ligation assays showed that NFκB-IκBα complexes were significantly increased in cells treated with the selinexor and bortezomib combination, further indicating that NFκB is inactivated by PI and XPO1 inhibitor treatment. Therefore, inhibition of NFκB by upregulating IκBα protein and subsequent creation of NFκB-IκBα complexes may be, at least in part, the mechanism behind proteasome inhibition and XPO1 inhibitor anti-tumor synergy. In evidence of this mechanism, we found that siRNA knockdown of IκBα significantly decreased the effect of selinexor, as shown by 10- to 25-fold increase in IC50 values, and knockdown significantly reduced selinexor/bortezomib-induced apoptosis. In addition selinexor/bortezomib treatment was shown to abrogate NFκB transcriptional activity.

In a companion report in this issue, Kashyap et al examined the combination of selinexor with PIs in sarcoma cell lines. They report that the combination treatment of selinexor with bortezomib sensitizes the sarcoma cells to the cytotoxic effects of PIs. In addition, this report highlights the importance of the NFκB signaling pathway in cancer, especially the role of the inhibition of NFκB or IκBα by selinexor/bortezomib treatment.

Selinexor, an orally active selective inhibitor of XPO1-mediated nuclear export (SINE), is currently undergoing phase I/II studies in a variety of indications, including a combination with carfilzomib, in both relapsed and refractory MM patients (NCT02199665). The results presented in this study support combinatorial clinical trials in relapsed and refractory MM that utilize PI therapies.

## MATERIALS AND METHODS

For human sample acquisition, written informed consent approved by an Institutional Review Board was obtained from all patients, in accordance with the Declaration of Helsinki. Patient samples were de-identified and obtained through the Institutional Review Board-approved Total Cancer Care® protocol at the Moffitt Cancer Center.

### Cell lines

Human MM cell lines RPMI 8226 (8226), IM-9, MM.1S, and U266 were obtained from the American Type Culture Collection (ATCC; Manassas, VA). To establish resistance of human MM cells to bortezomib, U266 and 8226 cells were continuously cultured in gradually increased concentrations of bortezomib. To produce PI-resistant U266 cells (U266PSR), bortezomib dosing started at 0.5 nM, increasing in step-wise increments of 0.2 nM to 20 nM [16, 19]. To produce PI-resistant 8226 (8226B25) cells, bortezomib dosing started at 1.0 nM, increasing in step-wise increments of 2.5 nM to 25 nM. The U266PSR cell line expressed a modest increase in Mcl-1, resulting in enhanced cell survival by inhibiting apoptosis and markedly lower expression of the apoptosis-promoting factor Bim [17]. Both of the bortezomib resistant cell lines, U226PSR and 8226B25, are also highly resistant to carfilzomib (see Figure 1).

U266PSR and 8226B25 cell lines were authenticated by the Moffitt Cancer Center Molecular Genomics Core Facility Cell lines using short tandem repeat (STR) DNA typing according to ATCC's “Authentication of Human Cell Lines: Standardization of STR Profiling (2012).” Results were compared with STR databases from ATCC and DSMZ (Deutsche Sammlung von Mikroorganismen und Zellkulturen, GmbH, Braunschweig, Germany) to establish percent identity. Cell lines were considered authenticated when the number of shared alleles across the eight core loci was ≥80% (as described by ATCC).

### Drug-resistant cell lines treated with XPO1 inhibitors and bortezomib or carfilzomib

Parental and drug-resistant human 8226, 8226B25, U266, and U266PSR MM cells were grown at low-density (log growth phase) conditions (3-4 ×10^5^ cells/mL) and cultured for 20 hours with either 300 nM selinexor (Karyopharm Therapeutics) or 10 nM KOS-2464 (Bristol-Myers Squibb) with and without 10 nM bortezomib (LC Labs), or 20 nM carfilzomib (SelleckChem). Optimal drug concentrations were determined by titration experiments for bortezomib and carfilzomib. Cells were fixed and permeabilized, and apoptosis was measured using anti-activated caspase 3/Alexa Fluor 488 (Cell Signaling Technology) staining in accordance with the manufacturer's standard protocol. Percent apoptosis was assayed by flow cytometry on a LSRII (Becton-Dickinson) bench-top analyzer. Data analysis was performed using Flowjo version 9.4 software (Tree Star, Inc) [24].

### NOD/SCID-γ mouse studies with selinexor ± bortezomib

All mouse studies were reviewed and approved by the Institutional Animal Care and Use Committee (IACUC), Research Integrity & Compliance - Research & Innovation at the University of South Florida. Bortezomib-resistant U266PSR human myeloma cells (10^6^) were injected subcutaneously into flanks of female NOD/SCID-γ mice, and tumors were allowed to grow for 14 days before the start of treatment [19]. U266PSR human myeloma tumors were treated twice weekly by intraperitoneal injection with bortezomib (0.5 mg/kg) or twice weekly by oral gavage with selinexor (10 mg/kg) or in combination where selinexor treatment was followed 2 to 3 hours later by bortezomib injection. Five mice were used per experimental group. Tumors were measured by calipers, and tumor volumes (mm^3^) were calculated by the formula (length x width^2^)/2. Mice were euthanized when a tumor volume exceeded 2000 mm^3^ or if the mouse lost >15% of its body weight. Drug toxicity was also assayed by mouse weights with a decrease of ≥10% considered an indication of toxicity by the drug regimen.

### Bone marrow aspirate processing and apoptosis assay of patient myeloma cells

As previously described, bone marrow aspirates were collected from newly diagnosed (n=8), relapsed (n=5), and PI-refractory bortezomib (n=8)/carfilzomib (n=6) patients [5]. PI refractory is defined as patients who progress during treatment or within 60 days after stopping treatment with either bortezomib or carfilzomib. Bone marrow aspirates (20 mL) from patients were isolated by Ficoll-Paque Plus (Amersham Biosciences) [5]. Cytospin slides were used to determine the percent plasma cell population by the microscopic morphology of toluidine-stained cells [5].

Isolated bone marrow mononuclear cells from the Ficoll-Paque fraction described above were also incubated at 4 × 10^6^/mL in 200 μL RPMI (Fisher) containing 10% FBS in 96-well plates, treated with either 300 nM selinexor or KOS-2464 with and without 10 nM bortezomib or 20 nM carfilzomib and incubated for 20 hours in a 5% CO_2_ humidified incubator. The following day, the cells were fixed and assayed for caspase activation-mediated apoptosis. Bone marrow mononuclear cells were fluorescently labeled with antibodies against activated caspase 3 (ASP175-Alexa 488), CD138 (M115-Alexa 647, BD Bioscience), and light-chain kappa (G20-193 V450, BD Bioscience) or lambda (JDC-12 V450, BD Bioscience). A BD Bioscience LSRII flow cytometer was used to gate MM cell populations, defined as cells that are both CD138 positive and light-chain positive. Non-myeloma patient bone marrow cells were defined as cells that were both CD138 and light-chain negative. Percent of apoptotic cells from each sample was assayed by activated caspase 3. Data analysis was performed using Flowjo version 9.4 software (Tree Star, Inc).

### Proximity ligation assay

Log-phase MM cells, both parental and PI-resistant, were placed at cell densities of 4 × 10^6^ cells/mL and treated with 300 nM selinexor for 4-6 hours. Cells were washed with PBS, and cytospins were made with 10^5^ cells/slide and fixed with 4% paraformaldehyde. Cells were incubated with primary antibodies to IκBα (E130/Abcam) and NFκB (L8F6/Cell Signaling). Incubation with the primary antibodies was followed by secondary antibodies conjugated with oligonucleotides provided in the Duolink kit (Olink Bioscience, Uppsala, Sweden) [25]. A red fluorescent signal was generated only when IκBα and NFκB, were in close proximity (<40 nm). 4', 6-Diamidino-2-phenylindole (DAPI) was used to stain the nuclei. Samples were observed with a Leica TCS SP5 AOBS laser scanning confocal microscope through a 63X/1.4NA Plan Apochromat oil immersion objective lens (Leica Microsystems CMS GmbH, Germany). We applied 405 diode, Argon 488, and HeNe 647 laser lines to excite the samples, with tunable emissions used to minimize crosstalk between fluorochromes. Z stack (0.5-μm-thick slices) images for each sample were captured with photomultiplier detectors, and maximum projections were prepared with the LAS AF software version 2.6 (Leica Microsystems).

Maximum projection images were analyzed using the Definiens® Developer v2.0 (Definiens AG, Munich, Germany) software suite. First, nuclei were segmented with an auto-threshold segmentation on the DAPI stain. Next, cytoplasms were segmented on the cellular auto-fluorescence in the green channel by using both size and intensity thresholds. Finally, the total number of foci per nucleus and cytoplasm were analyzed for number and area. This experiment was repeated 3 times. Western blots were made of the treated cells at 4-6 hours for XPO1 expression (according to the methods described below).

### Immunofluorescence microscopy and Western blot of IκBα in selinexor/bortezomib-treated drug-resistant MM cells and selinexor-treated patient MM cells

Drug-resistant 8226B25 and U266PSR human MM cells were incubated with selinexor (300 nM), bortezomib (10 nM), or their combination for 20 hours to determine their combined effects on IκBα expression by Western blot and immunofluorescence microscopy.

Western immunoblotting was performed as previously described [5]. Briefly, 100 μg of protein were loaded into each lane of an 8% SDS-PAGE gel (Bio-Rad) and transferred to PVDF membranes (Amersham) overnight (30 V at 4°C) with the use of a Bio-Rad Mini-Transblot apparatus. Membranes were blocked, and proteins were identified by incubation with specific antibodies: anti-IκBα (ab32518, Abcam) and GAPDH (clone 6C5 Millipore). Additional Western blots were performed to measure NFκB-mediated protein expression of anti-IAP-1 (Fisher PAS-29085), anti-IAP-2 (Fisher PAS-22997), anti-Myc (Fisher MAI-980), and anti-cyclin D2 (Fisher MA5-12731). All antibodies were used at a 1:1000 dilution in blocking buffer (5% instant non-fat dry milk in PBS) for 1 hour at ambient temperature. Membranes were washed, incubated with the appropriate secondary IgG-horseradish peroxidase, and visualized by enhanced chemiluminescence (Amersham).

Immunofluorescence microscopy of cytospins slides was performed as previously described [5] using both PI-resistant and parental cell lines and on patient MM samples (n = 6) after treatment with 300 nM selinexor, 10 nM bortezomib, or in combination. Anti-IκBα rabbit monoclonal antibody (Abcam) was used at 1:100 followed by incubation with anti-rabbit Alexa Fluor 594 (Invitrogen) secondary antibody (1:500). In addition patient bone marrow aspirate cytospins were stained with anti-kappa (Millipore AP505F) or anti-lambda (Millipore AP506F) light-chain FITC-conjugated antibodies to identify MM cells. Slides were washed four times in PBS, air dried, and covered with cover glass and Vectashield mounting media containing antifade/DAPI (Vector Laboratories Inc) to stain nuclei. Images were captured with a high-resolution CCD camera mounted on a Zeiss Automated Upright Fluorescent Microscope.

### ImageStream flow cytometry

The 8226B25 cell line was treated with 300 nM selinexor, 10 nM bortezomib, or selinexor/bortezomib and incubated for 20 hours. Cells were washed once in cold PBS and fixed for 20 minutes on ice in cytofix/cytoperm (BD Bioscience) solution. Cells were then pelleted and resuspended in perm/wash (BD Bioscience) solution and stored at 4°C. Treated cells (5 x10^5^) and controls were incubated for 1 hour at room temperature with anti IκBα (E130) (Abcam ab32518) diluted 1:50 in perm/wash (BD Bioscience) solution. Cells were then washed in perm/wash solution and incubated with anti-rabbit Alexa-488 diluted 1:250 and incubated for 45 minutes in the dark at room temperature. DAPI (Sigma D21490) (5 nM) was added to stain the nucleus immediately before analyses. Flow cytometry was performed on an ImageStreamX MKII high-speed imaging flow cytometer (Amnis Corporation) to analyze intracellular fluorescence. Bright field and fluorescent images were collected at a ×40 magnification. We assayed 10,000 gated cell singlets from each sample. IDEAS Analysis Software (Amnis Corporation) was used to determine nuclear location of IκBα in the treated and untreated cells. The Similarity Feature was used to determine nuclear location. Similarity is the log-transformed Pearson correlation coefficient and is a measure of the degree to which two images are linearly correlated within a designated region. This analysis uses the pixel data in the region that is specified as “the nucleus” (DAPI) and compares the similarity when other fluorochromes (IκBα) occupy the same pixel space. A high positive similarity value indicates that the probes are in the same location, whereas a high negative value indicates they are in different locations. A value near zero indicates there is an equal amount of probe in both the nucleus and the cytoplasm. Values were obtained for percent IκBα in the nucleus and cytoplasm in MM cells in all drug treatment groups.

### IκBα siRNA knockdown and its effect on selinexor IC_50_, apoptosis, and NFκB transcriptional activity

IM-9 and 8226 cell lines were transfected with 40 nM of IκBα siRNA or 40 nM BLOCK-iT control using Neon Transfection System (#MPK5000, Life Technologies) following manufacturer's instruction. The transfection was performed using antibiotic-free RPMI 1640 media. Twenty-four hours after transfection, the transfected and nontransfected IM-9 and 8226 cells were treated with selinexor starting at 30 μM and diluted 1:3 to a final concentration of 4.5 nM in triplicate for 72 hours. The cell viability was analyzed using CellTiter-Fluor cell viability assay (#G6080, Promega) and the half-maximal inhibitory concentration (IC_50_) of selinexor for each condition was calculated using XLfit. IκBα knockdown was 60% compared with transfection control. We transfected 8226, 8226B25, and H929 human MM cells with control siRNA or IκBα siRNA followed by treatment 48 hours after transfection with selinexor 300 nM, bortezomib 10 nM, and the combination. After 20-hour incubation with drugs, the cells were assayed by flow cytometry for activated caspase 3 apoptosis as above (n=2).

NFκB transcriptional activity was assayed in MM.1S cells pretreated with 1 μM selinexor and/or 100 nM bortezomib for 2 hours and then exposed to 20 ng/mL TNFα for 4 hours in serum-free media. TNFα exposure induced NFκB transcriptional activity 6-fold as measured by a chemiluminescent transcription factor assay kit (Thermo Scientific, catalog no. 89859). TNFα-activated NFκB p65 transcription factor binds to a biotinylated consensus sequence plated on a 96-well plate. The plate is developed using a primary antibody to NFκB p65 followed by a horseradish peroxidase-conjugated secondary antibody and developed with a chemoluminescent substrate.

### Statistical analysis

Analysis of variance was used to compare mouse data sets. All other statistical comparisons were made using the *t* test.
